# Testing indoor residual spraying coverage targets for malaria control, Bioko, Equatorial Guinea

**DOI:** 10.2471/BLT.24.292505

**Published:** 2025-05-03

**Authors:** Guillermo A García, Dianna E B Hergott, David S Galick, Olivier Tresor Donfack, Liberato Motobe Vaz, Lucas O Nze Nchama, Jeremías N Mba Eyono, Restituto M Nguema Avue, Matilde Riloha Rivas, Marcos M Iyanga, Faustino E Ebang Bikie, Teresa A Ondo Mifumu, Wonder P Phiri, Michael E von Fricken, Robert C Reiner, David L Smith, Carlos A Guerra

**Affiliations:** a MCD Global Health, 8403 Colesville Road, Silver Spring, MD 20910, United States of America (USA).; b Institute for Health Metrics and Evaluation, University of Washington, Seattle, USA; c Department of Health Metrics Sciences, University of Washington, Seattle, USA.; d MCD Global Health, Bioko Norte, Equatorial Guinea.; e Ministry of Health and Social Welfare, Bioko Norte, Equatorial Guinea.; f Department of Environmental and Global Health, University of Florida, Gainesville, USA.

## Abstract

**Objective:**

To test 50% indoor residual spraying coverage (percentage of households sprayed) for non-inferiority against the recommended 80% coverage for malaria control.

**Methods:**

Indoor residual spraying was done in 2021 and 2022 on Bioko, Equatorial Guinea, in a control arm (80% coverage) and intervention arm (50% coverage) with 37 clusters each. We assessed malaria infection in a representative sample of the population during annual surveys using rapid diagnostic tests. We compared the change in the odds of *Plasmodium falciparum* infection between baseline and post-intervention using difference-in-differences analysis within a survey-weighted binomial generalized linear model. Given differences between the arms at baseline, we adjusted the model for indoor residual spraying coverage at baseline.

**Findings:**

Relative to baseline, the odds of malaria infection post-intervention were 1.11 (95% confidence interval, CI: 0.81–1.52) in the 80% arm and 0.97 (95% CI: 0.72–1.29) in the 50% arm. In the adjusted model, the change in the odds of *P. falciparum* infection was no greater in the intervention arm than in the control arm (odds ratio: 0.89; 95% CI: 0.58–1.36), with the upper CI being lower than the non-inferiority margin of 1.43.

**Conclusion:**

There was no evidence that 50% coverage was inferior in preventing malaria, which supports the use of this target in settings where this level makes indoor residual spraying feasible by increasing the cost–effectiveness and equity of the intervention.

## Résumé

**Objectif:**

Évaluer l’infériorité de la couverture de 50% de pulvérisation intradomiciliaire d’insecticide à effet rémanent (pourcentage de ménages pulvérisés) par rapport à la couverture recommandée de 80% dans la lutte contre le paludisme.

**Méthodes:**

Des pulvérisations intradomiciliaires à effet rémanent ont été effectuées en 2021 et 2022 à Bioko, en Guinée équatoriale, dans un groupe témoin (couverture de 80%) et un groupe d’intervention (couverture de 50%), chacun comptant 37 grappes. Nous avons utilisé des tests de diagnostic rapide pour évaluer l’infection palustre dans un échantillon représentatif de la population lors des enquêtes annuelles. Nous avons comparé l’évolution de la probabilité d’infection par *Plasmodium falciparum* entre la situation de départ et celle postérieure à l’intervention à l’aide d’une analyse de l’écart des différences dans le cadre d’un modèle linéaire généralisé binomial pondéré par enquête. Compte tenu des différences entre les deux groupes au début de l’étude, nous avons ajusté le modèle en fonction de la couverture de pulvérisation intradomiciliaire d’insecticide à effet rémanent.

**Résultats:**

Par rapport à la situation de départ, la probabilité d’infection palustre après l’intervention était de 1,11 (intervalle de confiance (IC) à 95%: 0,81–1,52) dans le groupe à 80% et de 0,97 (IC à 95%: 0,72–1,29) dans le groupe à 50%. Le modèle ajusté ne présentait pas de variation plus importante de la probabilité d’infection à *P. falciparum* dans le groupe d’intervention par rapport au groupe témoin (odds ratio: 0,89; IC à 95%: 0,58–1,36), l’IC supérieur étant inférieur à la marge de non-infériorité de 1,43.

**Conclusion:**

Rien ne prouve que la couverture de 50% soit inférieure pour la prévention du paludisme, ce qui plaide en faveur de l’application de cet objectif dans les contextes où ce niveau permet la pulvérisation intradomiciliaire d’insecticide à effet rémanent en augmentant le rapport coût-efficacité et l’équité de l’intervention.

## Resumen

**Objetivo:**

Evaluar la no inferioridad de una cobertura del 50% de rociamiento intradomiciliario (porcentaje de hogares fumigados) en comparación con la cobertura recomendada del 80% para el control del paludismo.

**Métodos:**

Se realizó rociamiento intradomiciliario en 2021 y 2022 en la isla de Bioko, Guinea Ecuatorial, en dos grupos del estudio: un grupo de control (80% de cobertura) y un grupo de intervención (50% de cobertura), cada uno con 37 conglomerados. Se evaluó la infección palúdica en una muestra representativa de la población durante encuestas anuales, utilizando pruebas diagnósticas rápidas. Se comparó el cambio en la probabilidad de infección por *Plasmodium falciparum* entre el momento basal y la etapa posintervención mediante un análisis de diferencias en diferencias dentro de un modelo lineal generalizado binomial ponderado por encuesta. Dadas las diferencias observadas entre los grupos en el momento basal, el modelo se ajustó teniendo en cuenta la cobertura de rociamiento intradomiciliario inicial.

**Resultados:**

En comparación con el momento basal, la probabilidad de infección por paludismo en la etapa posintervención fue de 1,11 (intervalo de confianza [IC] del 95%: 0,81-1,52) en el grupo del 80% y de 0,97 (IC del 95%: 0,72-1,29) en el grupo del 50%. En el modelo ajustado, el cambio en la probabilidad de infección por *P. falciparum* no fue mayor en el grupo de intervención que en el grupo de control (razón de posibilidades: 0,89; IC del 95%: 0,58-1,36), siendo el límite superior del intervalo de confianza inferior al margen de no inferioridad de 1,43.

**Conclusión:**

No se encontró evidencia de que la cobertura del 50% fuera inferior para la prevención del paludismo, lo que respalda el uso de este objetivo en contextos donde dicho nivel de cobertura permite que el rociamiento intradomiciliario sea factible, mejorando la relación coste-eficacia y la equidad de la intervención.

## ملخص

الغرض اختبار تغطية بقايا الرش في الأماكن المغلقة بنسبة %50 (نسبة المنازل التي تم رشها) للتأكد من عدم وجود نقص مقارنةً بالتغطية الموصى بها والبالغة %80 لمكافحة الملاريا.

الطريقة تم تنفيذ بقايا الرش في الأماكن المغلقة في بيوكو، غينيا الاستوائية، خلال عامي 2021 و2022، في قسمي التحكم (تغطية %80)، والتدخل (تغطية %50)، مع 37 مجموعة لكل منهما. قمنا بتقييم عدوى الملاريا في عينة تمثيلية من السكان خلال مسوحات سنوية باستخدام اختبارات تشخيصية سريعة. قمنا بقارنة التغير في احتمالات الإصابة بالمتصورة المنجلية بين خط الأساس وما بعد التدخل باستخدام تحليل الفرق في الفروق ضمن نموذج خطي ثنائي معمم مرجح بالمسح. ونظرًا للاختلافات بين القسمين عند خط الأساس، قمنا بضبط نموذج تغطية بقايا الرش للأماكن المغلقة عند خط الأساس.

النتائج مقارنةً بخط الأساس، كانت احتمالات الإصابة بالملاريا بعد التدخل 1.11 (بفاصل ثقة مقداره %95: 0.81 إلى 1.52) في مجموعة %80، و0.97 (بفاصل ثقة %95: 0.72 إلى 1.29) في مجموعة %50. في النموذج المُعدَّل، لم يكن التغير في احتمالات الإصابة بالمتصورة المنجلية أكبر في مجموعة التدخل منه في مجموعة التحكم (نسبة الاحتمالات المعدلة:  0.89؛ ‏بفاصل ثقة مقداره %95: 0.58 إلى 1.36) مع كون الحد الأعلى لفاصل الثقة أقل من هامش عدم النقص البالغ 1.43.

الاستنتاج لم يكن هناك دليل على أن تغطية %50 كانت أقل فعالية في الوقاية من الملاريا، مما يدعم استخدام هذا الهدف في البيئات التي يجعل فيها هذا المستوى بقايا الرش الداخلي ممكنًا من خلال زيادة فعالية التكلفة والإنصاف في التدخل.

## 摘要

**目的:**

为了检测 50% 的室内滞留喷洒覆盖率（已进行滞留喷洒处理的家庭所占百分比）在疟疾防治方面的效果并不逊色于推荐的 80% 的覆盖率。

**方法:**

于 2021 年和 2022 年在赤道几内亚比奥科岛分别对对照组（80% 的覆盖率）和干预组（50% 的覆盖率）进行了室内滞留喷洒处理（以上两组各包含 37 个群组）。我们在年度调查期间通过快速诊断检测对具有代表性群体的疟疾感染情况进行了评估。我们在调查加权二项广义线性模型中运用双重差分分析对与恶性疟原虫感染几率变化有关的基线数据与干预后相关数据进行了对比。如果对照组与干预组的基线数据存在差异，则我们需针对室内滞留喷洒覆盖率的基线数据调整该模型。

**结果:**

相对于基线数据，覆盖率为 80% 的对照组在干预后的疟疾感染几率为 1.11（95% 置信区间 (CI)：0.81–1.52），而覆盖率为 50% 的干预组的感染几率则为 0.97（95% CI：0.72–1.29）。在调整后的模型中，干预组的恶性疟原虫感染几率变化并不比对照组的更加明显（比值比：0.89；95% CI：0.58–1.36），且 CI 上限值低于 1.43 的非劣效性界值。

**结论:**

目前并无证据表明 50% 的覆盖率在预防疟疾方面的效果较差，这就证明可在通过提高干预措施的成本效益和公平性来推动实施 50% 室内滞留喷洒覆盖率的地区采用这一指标。

## Резюме

**Цель:**

Провести сравнительный анализ 50%-го и рекомендуемого 80%-го охвата обработки помещений (доли обработанных распылением домохозяйств) инсектицидами остаточного действия для оценки их эффективности в борьбе с малярией.

**Методы:**

На протяжении 2021 и 2022 годов на о. Биоко (Экваториальная Гвинея) в помещениях проводилось распыление инсектицидов остаточного действия в контрольной группе (80%-й охват) и в группе вмешательства (50%-й охват), при этом в каждую группу было включено 37 кластеров. Авторы оценили количество случаев малярии в репрезентативной выборке популяции в ходе ежегодных опросов с использованием быстрых диагностических тестов. Для сравнения изменений вероятности инфицирования паразитом *Plasmodium falciparum* между исходным уровнем и периодом после распыления инсектицида использовался метод разностей в рамках обобщенной биномиальной линейной модели с учетом весовых коэффициентов, определенных на основании опросов. Авторы учли различия между группами, существовавшие на исходном уровне, и соответственным образом откорректировали модель охвата распыления инсектицидов остаточного действия в помещениях на этом уровне.

**Результаты:**

Вероятность инфицирования малярией после распыления в сравнении с исходным уровнем составила 1,11 (95%-й доверительный интервал, ДИ: 0,81–1,52) в группе 80%-го охвата и 0,97 (95%-й ДИ: 0,72–1,29) в группе 50%-го охвата. В скорректированной модели изменение вероятности инфицирования *P. falciparum* для группы вмешательства было не выше такового в контрольной группе (отношение шансов: 0,89; 95%-й ДИ: 0,58–1,36), при этом верхний предел ДИ был ниже предельного значения для нехудшего сравнительного результата, которое составляло 1,43.

**Вывод:**

Авторы не выявили значимых различий в эффективности 50%-го охвата в профилактике малярии, что подтверждает целесообразность использования этого целевого значения в тех условиях, где такой охват является осуществимым, тем самым повышается экономическая эффективность обработки и доступность вмешательства для всех слоев населения.

## Introduction

Indoor residual spraying has been widely used as a key malaria intervention. Early indoor residual spraying campaigns with dichlorodiphenyltrichloroethane contributed to the rapid elimination of malaria from the last areas in southern United States of America.^1^ This spraying was also credited for substantial reductions in malaria in other countries before and during the Global Malaria Eradication Programme (1955–1969).^2^^–^^4^ Although large-scale indoor residual spraying at the time was limited across most of Africa,^5^ pilot projects showed significant reductions in malaria transmission^4^^,^^6^ and provided evidence for scaling up the intervention in the 21st century.^7^

Indoor residual spraying is most beneficial when applied to multiple neighbouring households. Coverage, that is the percentage of households sprayed in a given area, is key to achieve community-wide protection. Universal coverage with indoor residual spraying, where all households in a target population are sprayed, was the original aim for malaria control programmes,^8^ but this target is unrealistic.

Indoor residual spraying is expensive and costs often prevent universal coverage. The implementation costs are due to the logistics of indoor residual spraying,^9^^,^^10^ including substantial investment in personnel and equipment.^11^ Additionally, insecticides are expensive, representing up to 50% of the overall cost.^11^^,^^12^ This cost has increased since 2012, when, to preserve the efficacy of pyrethroid-based insecticidal nets, the World Health Organization (WHO) called for phasing out the relatively inexpensive pyrethroid insecticides.^13^ The use of new formulations has led to an almost tenfold increase in cost of indoor residual spraying,^12^ which many countries cannot afford.^7^ Thus, there is a need to re-examine recommendations that can improve their feasibility.

One approach is to optimize coverage targets that use spillover effects, where people who do not receive the intervention still benefit from its effects.^14^ Given spillover, less than 100% coverage would still protect people whose houses were not sprayed, improving the cost–effectiveness of the intervention.^14^ Under this premise, WHO recommends that the indoor residual spraying coverage target be at least 80%.^15^ However, the basis of this recommendation is unclear.

Evidence shows the effect of indoor residual spraying on the risk of acquiring malaria, although few high-quality trials allow measuring effect sizes,^16^^,^^17^ and these trials have only evaluated spraying at high coverage (≥ 80%).^17^ Observational data provide mixed results of the effect of different levels of coverage. Data from Equatorial Guinea showed a decrease in the risk of malaria infection in individuals inhabiting an area with indoor residual spraying coverage ≥ 80% compared with people living in areas with poor coverage (*<* 20%), but no effect for people living in areas with medium coverage (50–79%).^18^ However, in Malawi, the same study showed a decreased risk of infection in people in areas with 50–79% coverage relative to people in low-coverage areas (*<* 20%), but no significant protection was observed with higher coverage (≥ 80%).^18^ In Madagascar, in areas where malaria prevalence in children younger than 5 years of age ranged between 5% and 14%, a significant reduction in incidence was seen in areas with very high spraying coverage (86–90%) relative to areas with ≤ 85% coverage, although the observed effect was not robust to sensitivity analyses and disappeared at higher coverage (*>* 90%).^19^ In a high malaria transmission area of Zambia, a reduction of 4–5% was observed with each 10% increase in spraying coverage, depending on the season, but it is not clear if increasing coverage had diminishing returns on this effect.^20^

These studies mostly support the need for high coverage, but cannot recommend the optimal coverage to trigger community protection from spillover and eventually suppress transmission.^21^ Such information can help malaria programmes weigh the benefits and costs of indoor residual spraying and optimize resources while maximizing the effect of spraying.^10^

We examined indoor residual spraying coverage targets on Bioko, Equatorial Guinea, from 2020 to 2022. Between 2004 and 2014, the Bioko Island Malaria Elimination Project supported the National Malaria Control Programme of Equatorial Guinea in delivering yearly indoor residual spraying island-wide. From 2015 to 2020, only the highest transmission areas were sprayed, leaving large fractions of the population uncovered. By 2019, the gains in reducing malaria prevalence had stalled, suggesting that spraying more areas may again be needed. However, budget constraints made spraying the whole island at ≥ 80% coverage unfeasible. Our recent dose–response analyses of observational data from Bioko showed the indoor residual spraying spillover effect could occur at 30% coverage with no significant gains thereafter.^22^ This finding prompted us to investigate a lower coverage that better balanced impact and intervention equity. 

## Methods

### Study design

We conducted an operational study using two arms to test the non-inferiority of 50% indoor residual spraying coverage (intervention) against the WHO-recommended 80% coverage (control). The outcome was change in the odds of *Plasmodium falciparum* infection in the intervention arm relative to the control, expressed as odds ratios (OR). We followed the WHO reporting guidelines for operational research.^23^

### Setting

Bioko has a population of about 270 000, around 80% of whom live in Malabo, the country capital.^24^ Malaria transmission is perennial, although higher between June and December. Before malaria control, transmission rates were among the highest for any malaria-endemic setting.^25^ *Anopheles funestus* and *An. gambiae* sensu stricto were the main vectors, but were eliminated after indoor residual spraying was introduced in 2004.^26^^–^^29^ Currently, the main vector is *An. coluzzii* with a small contribution to transmission by *An. melas.* Despite considerable gains in the past 20 years,^26^^,^^27^^,^^30^^–^^32^ transmission remains stable across the island with occasional outbreaks.^32^

**Fig. 1:**
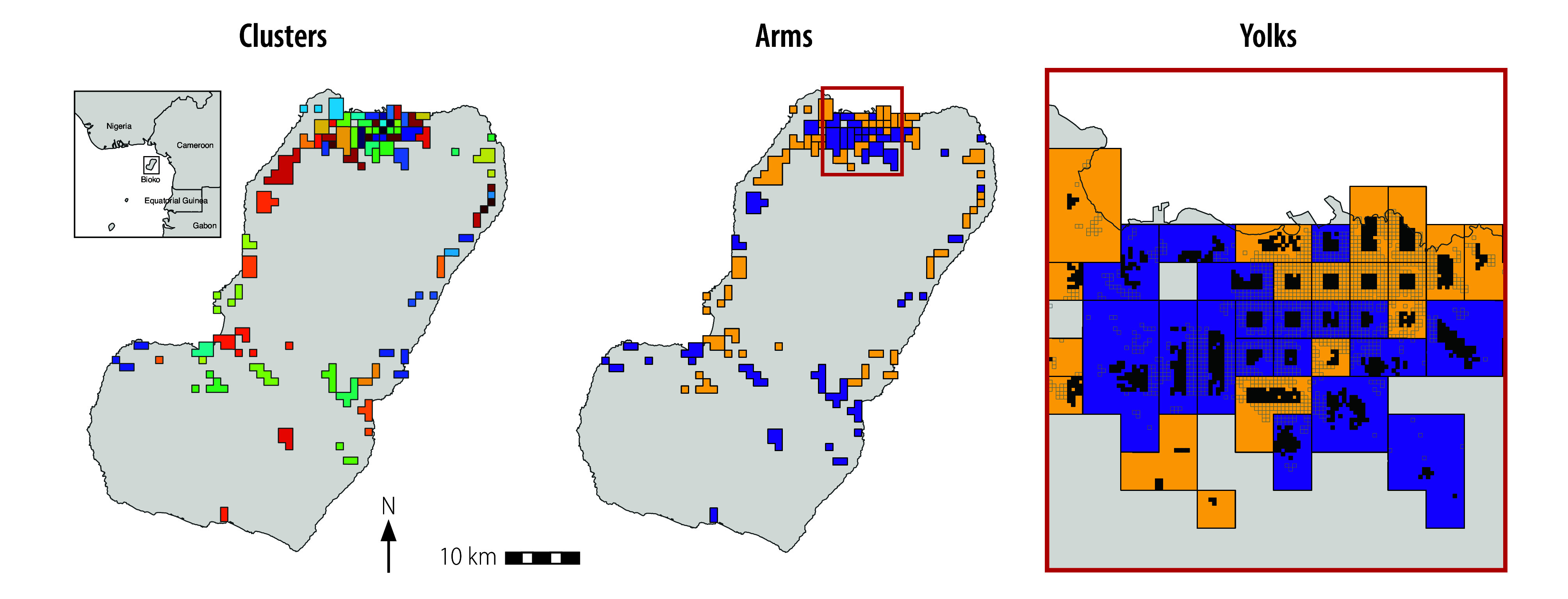
Distribution of clusters, arms and yolks, Bioko, Equatorial Guinea, 2021–2022

Notes: Clusters were based on primary sampling units of areas about 1 x 1 km. Populated, 100 x 100 m sectors (small squares in the right panel) received either 80% (purple) or 50% (yellow) spraying coverage. Cluster yolks, based on a distance of at least three sectors (300 m) from the closest sprayed sector in the neighbouring cluster, are shown in dark grey. The red box roughly corresponds to the city of Malabo.

**Fig. 2:**
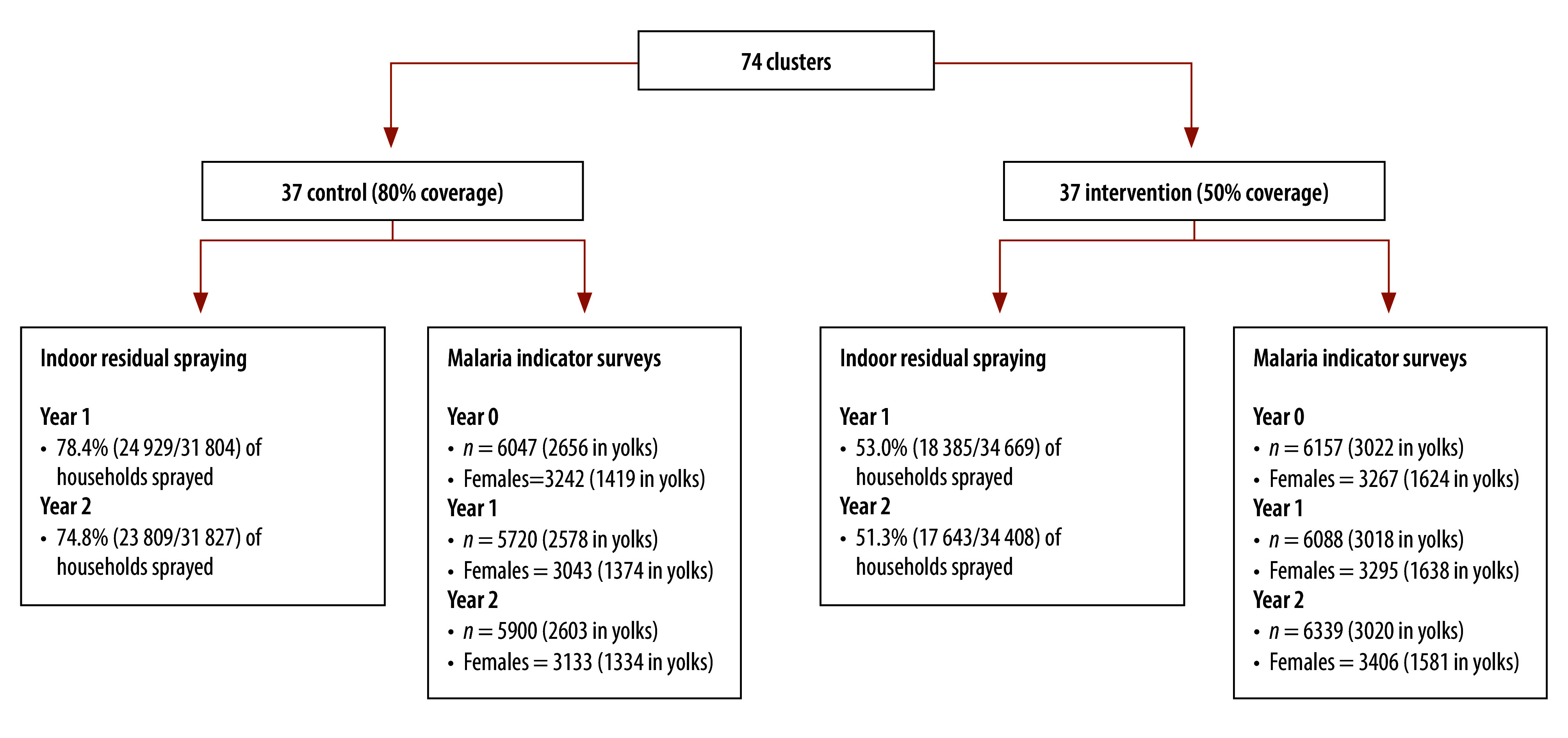
Households sprayed and malaria indicator surveys sample size by year, Bioko, Equatorial Guinea, 2021–2022

Notes: Percentages represent indoor residual spraying coverage. The number of female participants sampled by arm and within yolk is included for reference.

In 2021, we assigned almost all inhabited areas of Bioko to 74 clusters, randomly allocated (1:1) to control or intervention coverage (Fig. 1 and Fig. 2). Clusters were based on primary sampling units used in annual malaria indicator surveys, which were assigned to either an urban (34 clusters, 16 in control and 18 in intervention areas) or rural (40 clusters, 21 in control and 19 in intervention areas) stratum. We defined urban as having both high population density and relatively low residual transmission.^33^ We calculated the number of clusters based on a median number of 108 individuals per cluster at baseline (in 2020) and an estimated intracluster correlation coefficient of 0.09 with 80% power and α = 0.05. The number of clusters was influenced by the feasibility of spraying primary sampling units. Based on the above-mentioned calculations, 37 clusters per arm was enough to detect a change of 6–7% in *P. falciparum* parasite rate in the intervention group. We randomly assigned clusters and checked the number of households in each arm after each iteration. We chose the randomization that showed the best balance of spraying workloads to optimize deployment and facilitate implementation. We adopted a so-called fried-egg design, with the so-called yolk of each cluster located at least 300 m away from its border.^34^^,^^35^ We based analyses on data within these segregated areas to reduce the likelihood of contamination between control and intervention arms.

**Fig. 3:**
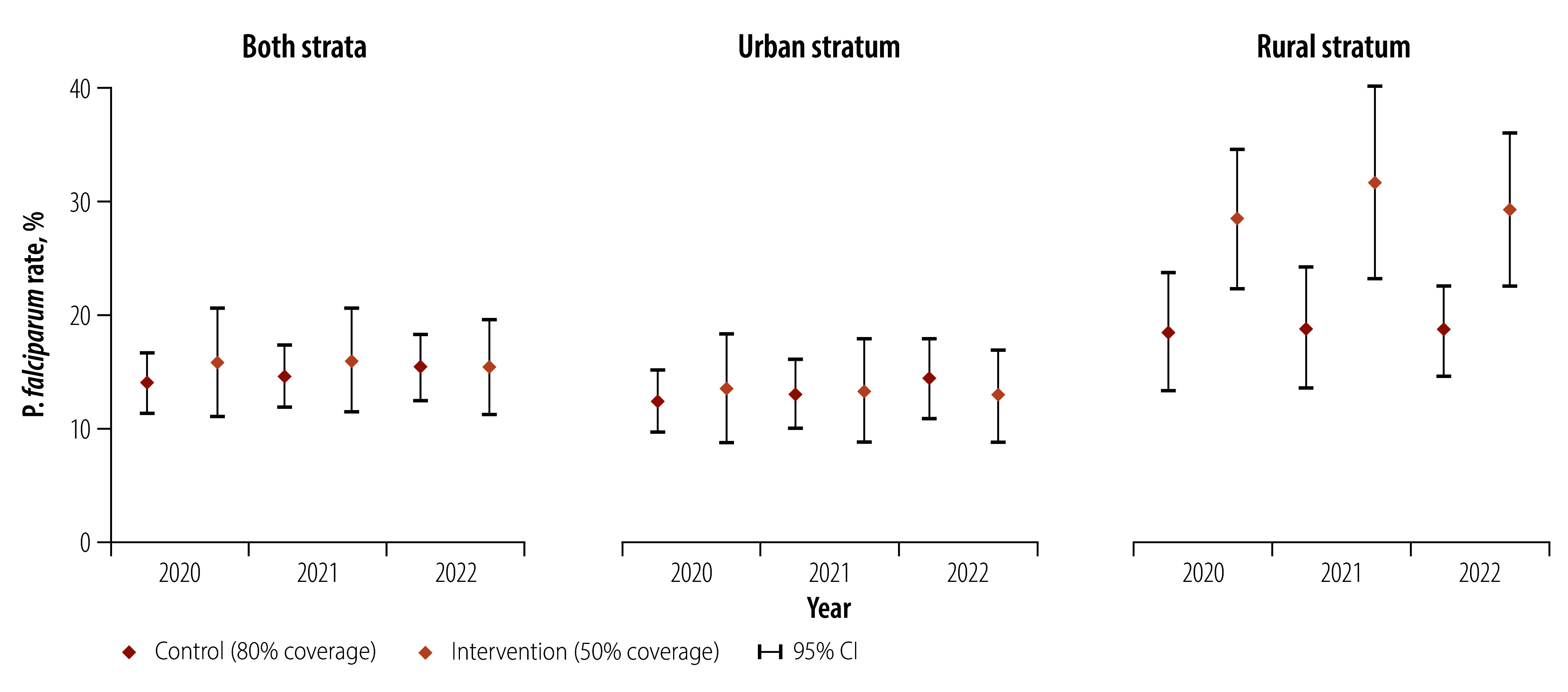
Mean *Plasmodium falciparum* parasite rate, by year and study arm, Bioko, Equatorial Guinea, 2021–2022

**Fig. 4:**
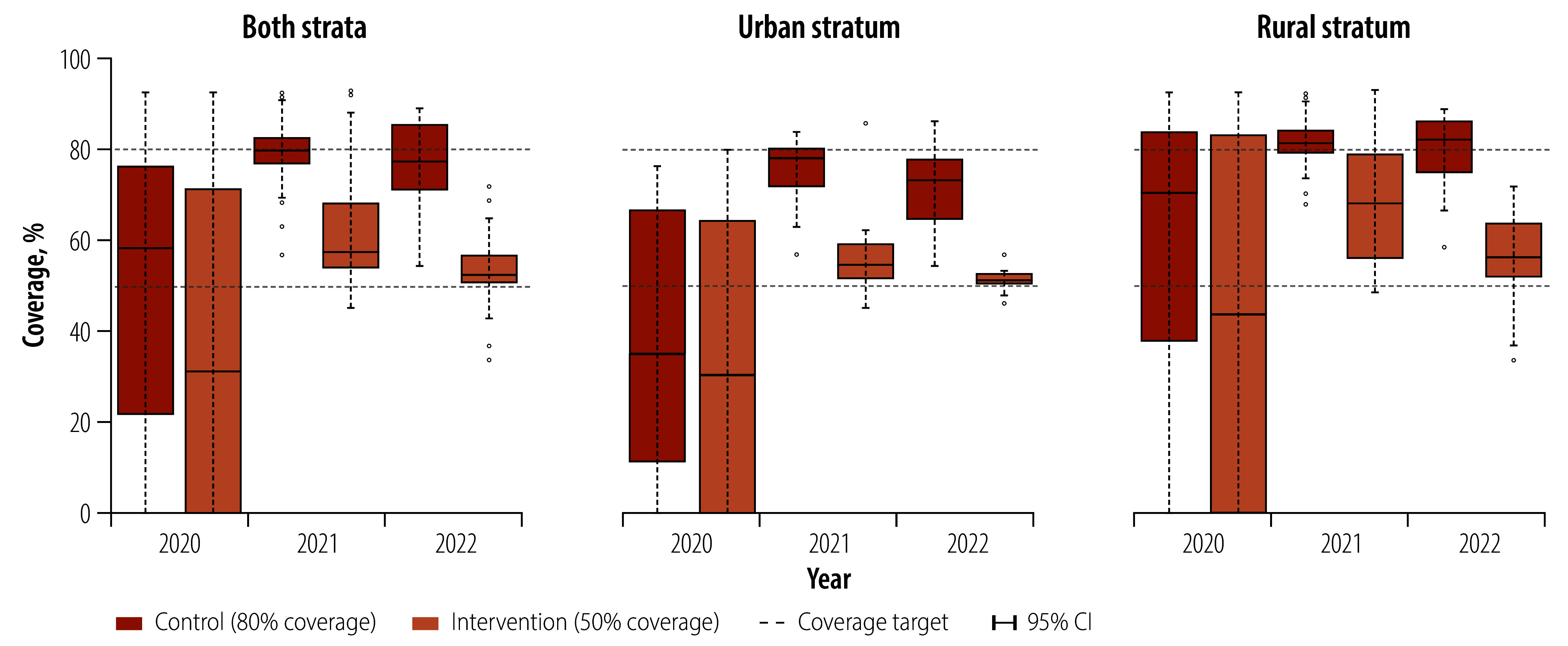
Cluster-level indoor residual spraying coverage, by year and study arm, Bioko, Equatorial Guinea, 2021–2022

Notes: The thick black line within each box is the median coverage and the lower and upper box bounds is the interquartile range. The range is represented by the horizontal lines of the whiskers and the empty circles show outliers or data points at least 1.5 times under or over the first and third quartiles.

We derived baseline data for indoor residual spraying coverage (percentage of households sprayed) and *P. falciparum* infection (as a binary variable) from the indoor residual spraying round and malaria indicator survey in 2020 (Table 1). We calculated the parasite rate as the proportion of *P. falciparum*-positive individuals. We balanced risk factors for malaria infection, including age, sex, bednet use and history of off-island travel,^36^ between arms. Variables were balanced at baseline, except the parasite rate (higher in the intervention arm in rural clusters; Fig. 3) and median spraying coverage (higher in the control arm; Fig. 4).

**Table 1:** Baseline characteristics of the study population, by study arm, Bioko, Equatorial Guinea, 2020

Characteristic	Control arm (80% coverage) (*n* = 6047)	Intervention arm (50% coverage) (*n* = 6157)
**Median age (range), years**	18 (1–92)	18 (1–104)
**Female participants, no. (%)**	3242 (53.6)	3267 (53.1)
***P. falciparum* parasite rate, % (95% CI)**		
Total	14.0 (11.3–16.6)	15.8 (11.0–20.6)
Females	12.6 (9.3–16.0)	14.6 (10.3–19.0)
Urban stratum	12.4 (9.7–15.1)	13.5 (8.7–18.3)
Rural stratum	18.5 (13.3–23.7)	28.4 (22.3–34.5)
**Slept under a net the previous night, no. (%)**	2386 (39.5)	2413 (39.2)
**Travelled to mainland, no. (%)**	111 (1.8)	110 (1.8)
**Inhabited houses, no.**	34 853	36 951
**Median indoor residual spraying coverage, % households sprayed**	57.8	31.2

CI confidence interval; *P*.: *Plasmodium*.

Notes: Data are based on the 2020 malaria indicator survey and indoor residual spraying round. Estimates of the parasite rate are weighted by malaria indicator survey design. The low rate of travel in 2020, relative to previous years,^33^ was due to coronavirus disease 2019 travel restrictions. The number of inhabited houses by arm varies year on year as the population tends to move houses frequently, or houses are built or destroyed.

CI: confidence interval.

### Implementation

We did two consecutive indoor residual spraying rounds (February–July 2021 and February–June 2022). We used the Bioko Island Malaria Elimination Project’s spatial decision support system^10^^,^^37^ for implementation of the study. We used 100 × 100 m sectors as the spatial units for deployment and coverage monitoring.^10^

We used a mix of non-pyrethroid insecticides according to available stock. In 2021, 43 314 houses were sprayed: 40 522 (93.6%) with clothianidin (SumiShield^®^ 50WG, Sumitomo Chemical Co. Ltd, Japan); 1941 with pirimiphos-methyl (4.5%; Actellic^®^ 300CS, Syngenta, Switzerland); and 851 (2.0%) with Fludora^®^ Fusion (Bayer S.A.S., France), a combination of clothianidin and deltamethrin. In 2022, 41 452 houses were sprayed using exclusively Fludora^®^ Fusion. Local insecticide resistance monitoring data showed mosquitoes remained highly susceptible to all insecticides used.

### Variables and data sources

We measured the primary outcome (*P. falciparum* infection) during malaria indicator surveys conducted in the rainy season (August and September) of the same years. In each survey, we used stratified cluster sampling of 5% of inhabited households in urban clusters and 25% in rural clusters to obtain an overall sample size about 5% of the island population (Fig. 2). We tested all consenting individuals in selected households for malaria infection (CareStart^™^ Pf/PAN (HRP2/pLDH) Ag Combo RDT, Access Bio, Somerset, USA). Individuals positive for malaria received a course of antimalarial medicines as per national guidelines.^38^ The parasite rate estimates presented here are survey-weighted estimates of individuals tested within the yolk of each cluster.

To determine spraying coverage, we calculated community coverage around each household (household-level coverage) as the fraction of homes sprayed within a 300 m radius of each household.^34^^,^^35^ We averaged household-level coverage for each cluster to provide cluster-level coverage and standardized for use in the models. We defined per protocol coverage as a range of 70–100% in the control and of 45–60% in the intervention arm, allowing for expected variability in coverage during implementation.

### Analyses

We used a difference-in-differences analysis to compare changes in odds of plasmodium infection post-intervention and between arms. We used a survey weighted binomial generalized linear model. The model contained three basic terms. First, there was an arm term representing the difference in odds of infection at baseline between the control and intervention arms. To investigate non-inferiority in a setting where there is perfect balance at baseline, we would expect this term to be close to 0. Second, we had a time term, reflecting the difference between the odds of infection in the control arm before (2020) and after (2022) the intervention. Third, we had a difference-in-differences term for the interaction between arm and time, thus providing an estimate of the difference in odds of infection over time between the intervention and control arms. We used the difference-in-differences term to estimate the effect size of the intervention relative to the control arm and its uncertainty to assess non-inferiority. With no difference between the intervention and control arms, the odds ratio of change in infection would be 1 (i.e. difference-in-differences term = 0). We used a non-inferiority margin of 1/0.7 = 1.43,^39^ meaning if the upper confidence interval (CI) was lower than this margin, we would conclude non-inferiority. Given differences in pre-intervention spraying coverage at baseline, we adjusted the model using a baseline coverage term corresponding to coverage in 2020 (Table 1).

We did secondary analyses of the impact of actual intervention coverage to account for deviations from the per protocol coverage range. Additionally, we explored the differences in impact by stratum and investigated the effect size at different levels of baseline coverage (online repository).^40^

We used R version 4.4.0 (R Foundation, Vienna, Austria) for analyses, and the survey package^41^ to generate survey weights according to the sampling strategy of malaria indicator surveys.

### Ethical considerations

This study was part of routine malaria control activities and operational research on Bioko. All interventions within the Bioko Island Malaria Elimination Project and National Malaria Control Programme of Equatorial Guinea, including vector-control activities, are approved by the Ministry of Health and Social Welfare of Equatorial Guinea through the presentation of annual work plans, which include ethical considerations.

The indoor residual spraying implementation strategy was approved within these routine work plans, with regular progress updates presented to the health ministry, donors and other stakeholders. The health ministry considered that the strategy was non-experimental as Bioko routinely undergoes changes in indoor residual spraying target areas as part of normal operations. No households were denied the intervention. As our study constituted operational research within routine malaria control activities rather than an intervention or cluster-randomized trial as defined by WHO and the scientific community, it was not registered in a clinical trials registry.

The malaria indicator surveys have ethical approval from the health ministry and the London School of Hygiene and Tropical Medicine granted at the start of the Bioko Island Malaria Elimination Project. As part of the protocol, all individuals found positive for malaria receive antimalarial treatment according to national guidelines.

While malaria control on Bioko is funded by a public–private partnership between the government and a consortium of oil companies, the funders had no influence on our study design and analyses. 

## Results

Fig. 2 summarizes the numbers of individuals sampled and the overall indoor residual spraying coverage by arm and year. Age and sex of the population sampled in 2021 and 2022 were similar to baseline (Table 1).

Table 2 and Fig. 3 give the mean survey weighted *P. falciparum* parasite rate measured before (2020), during (2021) and after (2022) the intervention. The rate was higher in the rural stratum and significantly so in the intervention arm, peaking in 2021. In the urban stratum, mean prevalence was not statistically different between arms and relative to the baseline.

**Table 2:** *Plasmodium falciparum* parasite rate and indoor residual spraying coverage in control and intervention arms, by stratum and year, Bioko, Equatorial Guinea, 2021–2022

Stratum	2021		2022	
	Control	Intervention	Control	Intervention
**Rural**				
Median indoor residual spraying coverage, % (IQR)	81.6 (79.1–83.9)	67.9 (56.4–78.9)	82.1 (75.0–86.1)	56.3 (52.0–63.5)
Mean parasite rate, % (95% CI)	18.8 (13.5–24.1)	31.7 (23.2–40.1)	18.5 (14.5–22.4)	29.2 (22.5–35.9)
**Urban**				
Median indoor residual spraying coverage, % (IQR)	78.0 (72.1–79.9)	54.8 (51.5–58.8)	73.0 (64.9–77.7)	51.3 (50.6–52.5)
Mean parasite rate, % (95% CI)	13.0 (10.0–16.0)	13.3 (8.8–17.8)	14.3 (10.8–17.8)	12.8 (8.8–16.8)

CI: confidence interval; IQR: interquartile range.

Note: Data were determined by the 2021 and 2022 malaria indicator surveys and indoor residual spraying rounds.

**Fig. 5:**
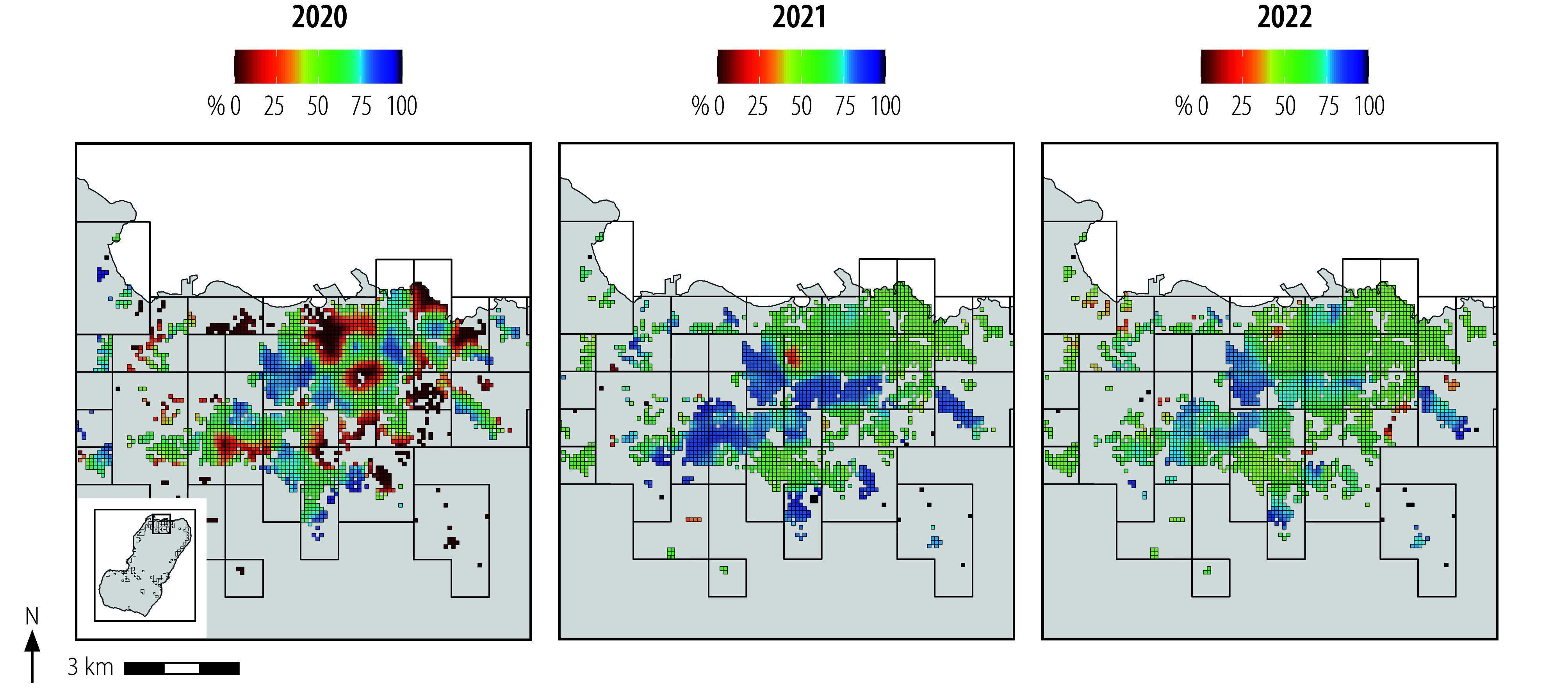
Indoor residual spraying coverage in urban Malabo, by year, Bioko, Equatorial Guinea, 2021–2022

Notes: The inset in the 2020 map shows the location of Malabo on Bioko. The lined areas are the clusters. Coverage is expressed as the average household-level coverage by sector using a 300 m buffer.

Fig. 4 shows the highly heterogeneous spraying coverage at baseline as a result of the targeting strategy; many clusters received no spraying, particularly in the intervention arm. During the study, only two clusters received < 40% coverage (33.7% and 37.9%, both in the intervention arm and the rural stratum in 2022), with coverage narrowly distributed, especially in the urban stratum (Fig. 4 and Fig. 5). Per-protocol coverage was achieved in many clusters, although it proved operationally challenging in others (Table 3). Coverage in the control arm was more easily attained in rural clusters, with little variation around the 80% mark, particularly in 2021. This coverage was harder to achieve in urban clusters, particularly in 2022, when only 11 of 16 clusters (68.8%) were within the per protocol coverage range. In contrast, intervention-arm coverage was easier to realize in urban clusters (Fig. 5), notably so in 2022 when all 18 clusters were within range (Table 3). In the rural stratum, many intervention clusters received more spraying than per protocol, especially in 2021, when only six of 19 clusters (31.6%) were within range and median coverage was 67.9% (Table 3 and Fig. 4).

**Table 3:** Clusters within range of per protocol coverage, by arm and year, Bioko, Equatorial Guinea, 2021–2022

Year	No. (%)	
	Control arm	Intervention arm
**2021**		
Rural	20/21 (95.2)	6/19 (31.6)
Urban	13/16 (81.3)	14/18 (77.8)
**2022**		
Rural	19/21 (90.5)	8/19 (42.1)
Urban	11/16 (68.8)	18/18 (100.0)

Note: We defined an acceptable range as between 70% and 100% for the control arm and between 45% and 60% for the intervention arm.

**Fig. 6:**
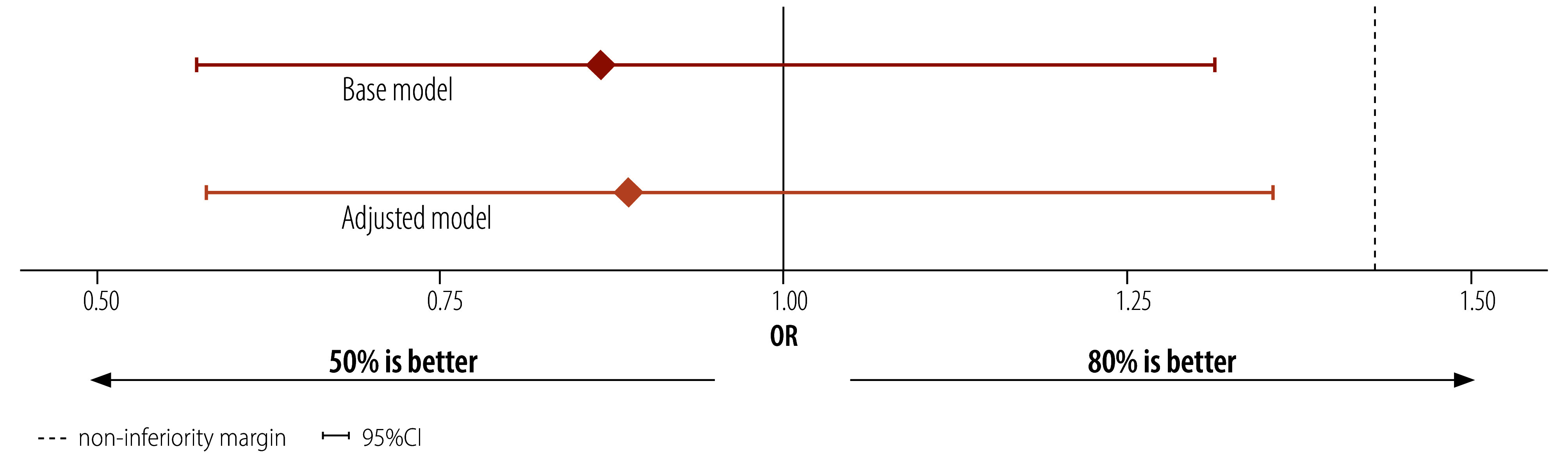
Difference-in-differences analysis of the effect of 50% versus 80% indoor residual spraying coverage on malaria infection odds, Bioko, Equatorial Guinea, 2020–2022

CI: confidence interval; OR: odds ratio.

Notes: The OR of the difference-in-differences term is illustrated with uncertainty.

The odds of malaria infection at the end of the study (2022) were not significantly different from baseline (2020) in both arms: 1.11 (95% CI: 0.81–1.52) in the control and 0.97 (95% CI: 0.72–1.29) in the intervention arms. The difference-in-differences term in both models was not significant: base model OR: 0.87 (95% CI: 0.57–1.31); adjusted model OR: 0.89 (95% CI: 0.58–1.36), with the 95% CI overlapping the no difference line and not exceeding the 1.43 non-inferiority margin (Fig. 6). We did additional model iterations and found no evidence of inferiority (online repository).^40^.

## Discussion

We compared the spillover effect of high (≥ 80%) versus medium (50%) indoor residual spraying coverage and found no significant difference in *P. falciparum* infection odds between the two arms, indicating no evidence of 50% coverage being inferior. In fact, the mean model estimates and lower CI were inclined towards superiority of 50% coverage. This finding can be explained by the lower baseline coverage in some intervention clusters compared with control clusters, particularly in urban areas, where receiving a medium coverage had a higher relative effect (online repository).^40^

The data in our study were collected and recorded at the household level and linked to a robust, error-controlled, geographically accurate spatial decision support system.^10^^,^^37^ As such, data were unambiguously assigned to a location, which eliminated recall bias when reporting house-spraying status. This system also allowed monitoring and guiding of spray teams, thus giving a precise representation of coverage. Furthermore, we conducted the study under real-world conditions in an operational context.

We originally aimed to use a non-inferiority, cluster randomized trial design. However, operational constraints resulted in two important limitations. First, differences existed in baseline spraying coverage as described earlier. To account for the differences, we adjusted the model for baseline coverage, although we observed no significant change in the impact of the intervention. The difference in baseline coverage could also partly explain the difference in baseline *P. falciparum* infection particularly noticeable in rural clusters. When running the base model separately in low baseline coverage communities (*<* 45%), the observed impact of the intervention leant even further towards superiority. Conversely, running the model in high baseline coverage communities (≥ 70%) inclined the effect towards inferiority, although the overall result was inconclusive (online repository).^40^ Second, while we aimed to achieve per protocol coverage, it would have been unethical not to spray in the intervention arm if someone wanted it. This situation resulted in many rural clusters in the intervention arm receiving higher than 50% coverage (Table 3). At the same time, spraying at 80% coverage in urban areas had challenges, including large numbers of houses to spray and higher refusal rates, resulting in suboptimal coverage in the control arm. In a sensitivity analysis, we adjusted the model with actual coverage achieved during the study as an intention-to-treat covariate but observed no difference in the modelled outputs (online repository).^40^ These limitations led to the use of the adjusted difference-in-differences binomial models described rather than standard *t*-tests used in cluster randomized trials.

Notably, on Bioko, *An. coluzzii* exhibits a mix of endo- and exophaghic activity. Nevertheless, recent analyses pairing local vector and human behaviours showed indoor biting is still driving most transmission, suggesting indoor residual spraying and long-lasting insecticidal nets are reducing transmission but not interrupting it.^42^

Our results were not likely confounded by environmental factors, notably rainfall, because the annual rounds of spraying, including the rounds reported here, are timed before the start of the main rains on Bioko. However, the impact of lower targets for indoor residual spraying coverage may differ depending on other local factors. Transmission intensity may influence the effect size of lower indoor residual spraying coverage. Similarly, insecticide resistance of local vectors could affect the optimal spraying coverage required. Another potential confounder is that Bioko is an island, although previous studies showed its high connectivity to mainland Africa through the movement of people.^33^^,^^43^ Investigation of lower targets for indoor residual spraying coverage is needed in other malaria-endemic settings, particularly where mosquito ecology, vector composition, malaria transmission and insecticide resistance differ.

Despite limitations for the generalizability of our findings, they have important implications for vector control. Achieving high indoor residual spraying coverage can be particularly challenging where the population is likely to refuse the intervention or where houses are difficult to access.^10^ High population density areas are especially hard to spray at high coverage due to the volume of houses. In Bioko, spraying budgeting and planning is based on four households sprayed per sprayer per day.^10^ Therefore, coverage of 80% in an urban sector with 78 houses requires 15.6 worker days, in contrast to 2.2 worker days in a rural sector with 11 households. Reducing the coverage target to 50% would equate to 9.8 worker days in urban and 1.4 worker days in rural sectors. Spraying urban Malabo is further complicated by high refusal and absenteeism, which slow productivity and require multiple visits. A lower coverage target in Malabo would reduce the problem of refusal. In contrast, spraying productivity in rural sectors is facilitated by easier access, lower refusal and higher demand from the population, so a lower target would have a lower impact. In absolute terms, lower spraying coverage translates into substantial cost and logistics savings in urban Malabo but less so in the rural periphery. This situation is probably true in other malaria-endemic areas and is especially important where financial support for malaria vector control is insufficient.

Other alternatives have been proposed to reduce the burden of indoor residual spraying, such as partial or selective spraying where only parts of the walls are sprayed.^44^ Much of the effort in spraying is house preparation, which is unaffected if walls are sprayed partially, unless it means restricting spraying to the upper parts of the walls and ceilings.^44^ More importantly, spray teams must access the house, so intervention refusal would still affect productivity and coverage. Therefore, this alternative does not fully address the main complexities of indoor residual spraying at scale, although it could be complementary in a scenario of lower spraying coverage targets for even higher intervention efficiency. Our study suggests the lower coverage approach is a plausible strategy to expand the intervention area and reach a higher fraction of the population at risk using the same resources, or to save resources that could be reallocated to other interventions. The National Malaria Control Programme of Equatorial Guinea, with evidence from this study, has already adjusted indoor residual spraying coverage targets, which is facilitated by the spatial decision support system.^10^ A similar approach could bring indoor residual spraying back as an option for malaria control in countries that have moved away from the intervention due to perceptions of ineffectiveness and challenging expectations set by WHO. While innovative interventions, such as vaccines and monoclonal antibodies, promise support for malaria control, they face efficacy and cost–effectiveness issues.^45^ Driving innovation in the delivery of proven interventions, such as indoor residual spraying, can help overcome current bottlenecks to reducing the malaria burden.

Our findings provide evidence for revising current indoor residual spraying coverage recommendations. While such spraying is an effective vector-control intervention, operational challenges can limit its feasibility. Given the evidence of non-inferiority, we urge stakeholders and decision-makers revisit current standards and coverage targets^15^ and promote cost–benefit analyses and further research in other settings to validate our findings.
